# Cardiovascular Biomarkers as a Primary Care Gateway to Early Alzheimer's Disease Detection: The Case for an Integrated Screening Approach

**DOI:** 10.7759/cureus.110287

**Published:** 2026-06-05

**Authors:** Aizhan Tuleubayeva

**Affiliations:** 1 Internal Medicine, Semey Medical University, Almaty, KAZ

**Keywords:** alzheimer's disease, cardiovascular biomarkers, c-reactive protein, dementia prevention, early detection, heart-brain axis, integrated screening, nt-probnp, primary care screening, p-tau217

## Abstract

Alzheimer's disease (AD) affects millions of Americans and represents one of the leading causes of disability and healthcare expenditure in the United States. The vast majority of patients are diagnosed at the symptomatic stage, when substantial neuronal loss has already occurred and the therapeutic window for disease-modifying treatment has closed. Recently approved disease-modifying therapies have created an urgent clinical need for pre-symptomatic patient identification. The cardiovascular risk factors most commonly managed in primary care -- hypertension, dyslipidemia, type 2 diabetes, atrial fibrillation, and chronic heart failure -- are among the most powerful modifiable antecedents of AD pathology, operating through systemic inflammation, cerebral small vessel disease, impaired glymphatic clearance, and tau hyperphosphorylation. The biomarkers used to monitor these conditions -- C-reactive protein, cardiac troponin, NT-proBNP, and homocysteine -- reflect active neurodegeneration risk processes already measured routinely in primary care. This clinical perspective proposes a three-stage integrated neuro-cardiological screening protocol linking cardiovascular biomarker assessment to plasma P-tau217 blood testing for AD confirmation. This framework addresses the implementation gap identified in recent United States primary care literature and represents a practical step toward closing the AD diagnostic gap.

## Introduction and background

Alzheimer's disease (AD) currently affects between 7.2 and 7.4 million Americans age 65 and older, with projections reaching 13.8 million by 2060 and annual healthcare costs reaching $384 billion [1]. The vast majority of patients are diagnosed at the symptomatic stage, when substantial neuronal loss has already occurred and the therapeutic window for disease-modifying intervention has closed [2]. This diagnostic gap has acquired new clinical urgency with the FDA approval of lecanemab and donanemab -- the first disease-modifying therapies for AD, both specifically indicated for early-stage, biomarker-confirmed patients per the 2024 revised NIA-AA diagnostic criteria [3-5]. Plasma phosphorylated tau at threonine 217 (P-tau217) fulfills the key biomarker criterion in this framework, with an area under the receiver operating characteristic curve exceeding 0.91 and a pre-symptomatic detection window of three to five years [6]. Commercial P-tau217 testing is now available through Quest Diagnostics and Labcorp following FDA Breakthrough Device Designation in 2024 [6].

Despite the availability of blood-based biomarker testing, a critical implementation gap persists. Primary care physicians in the United States view P-tau217 tests as accurate and cost-effective but lack an operational workflow for systematic patient identification and testing [7]. The cardiovascular risk factors most commonly managed in primary care -- hypertension, dyslipidemia, type 2 diabetes, atrial fibrillation, and chronic heart failure -- are among the most powerful modifiable antecedents of AD pathology [8]. The Lancet Commission on dementia (2024) identified vascular risk factors as responsible for a substantial proportion of preventable dementia cases [8]. The biomarkers used to monitor cardiovascular disease -- C-reactive protein (CRP), cardiac troponin, NT-proBNP, and homocysteine - reflect pathophysiological processes that directly contribute to the neurodegeneration cascade through four established mechanisms: systemic inflammation promoting blood-brain barrier disruption, cerebral small vessel disease (CSVD) impairing white matter integrity, glymphatic failure accelerating amyloid-beta accumulation, and tau hyperphosphorylation driven by vascular neuroinflammation [9,10].

The patient population at highest cardiovascular risk and the population at highest AD risk are, to a substantial degree, the same people -- already presenting to primary care physicians for routine cardiovascular management. This clinical perspective proposes a structured, three-stage primary care framework that systematically links cardiovascular biomarker assessment to P-tau217 blood testing for early AD detection, addressing the implementation gap identified in the published literature and translating established mechanistic evidence into a practical clinical workflow.

## Review

The heart-brain axis: mechanistic basis for cardiovascular biomarker screening

The mechanistic link between cardiovascular disease and AD pathology operates through four established pathways. First, chronic systemic inflammation -- reflected by elevated high-sensitivity CRP and interleukin-6 -- promotes vascular endothelial dysfunction, blood-brain barrier disruption, and cerebral hypoperfusion, accelerating amyloid-beta accumulation [9]. Second, CSVD, driven by hypertension and atrial fibrillation-related cerebral microemboli, disrupts white matter integrity and reduces cerebral reserve -- the critical intermediate mechanism between systemic vascular disease and cognitive decline [9]. Third, impaired glymphatic clearance -- consequent to reduced cardiac output, sleep-disordered breathing, and cerebral hypoperfusion -- allows neurotoxic amyloid-beta and tau metabolites to accumulate rather than being cleared during sleep [10]. Fourth, vascular neuroinflammation activates tau kinases (CDK5 and GSK-3beta) that produce the hyperphosphorylated tau tangles characteristic of AD pathology [5].

Homocysteine -- elevated in 30% to 40% of patients with AD -- reflects impaired folate and B12 metabolism and endothelial dysfunction and represents an independent risk factor for both cerebrovascular disease and AD [11]. Atrial fibrillation specifically has been identified as an independent risk factor for dementia and AD, operating through cerebral microembolism, reduced cardiac output, and AF-associated cerebral hypoperfusion - mechanisms that are directly captured by the NT-proBNP and cardiac troponin assessments proposed in Stage 2 of this framework [12].

The three-stage integrated neuro-cardiological screening protocol

The proposed framework is designed for deployment within existing primary care infrastructure, requiring no specialist referral until Stage 3 confirmation.

Stage 1 (cardiovascular risk screening): At annual preventive care visits for adults aged 50 and older, composite cardiovascular risk assessment using the Framingham risk score (threshold ≥10%) or metabolic syndrome criteria, combined with high-sensitivity CRP and optional plasma homocysteine. Patients meeting risk thresholds proceed to Stage 2.

Stage 2 (cardiac-cerebral bridge assessment): High-sensitivity cardiac troponin (hs-cTnI/T) and NT-proBNP to quantify cardiac microvascular compromise and cerebral perfusion impairment, combined with ECG for atrial fibrillation detection. Elevated markers trigger progression to Stage 3.

Stage 3 (AD biomarker confirmation): Plasma P-tau217 testing, commercially available via Quest Diagnostics and Labcorp [6]. Positive results trigger neurology referral for clinical confirmation and evaluation for disease-modifying therapy eligibility (lecanemab or donanemab). Optional adjunctive glial fibrillary acidic protein (GFAP) testing for ambiguous results.

The sequential design progressively narrows the population, warranting the most specific test and maximizing the positive predictive value of P-tau217 at Stage 3. At Stage 1, approximately 25% to 30% of screened adults aged 50 and older carry elevated cardiovascular risk. Of these, an estimated 8% to 12% will demonstrate Stage 2 cardiac-cerebral biomarker elevation. Of those reaching Stage 3, approximately 20% to 40% are expected to test P-tau217 positive, consistent with published amyloid positivity rates in high cardiovascular risk populations [6,7].

Clinical rationale and personal observation

The clinical rationale for this framework is grounded not only in published evidence but also in direct clinical observation. In nine years of practice as a cardiologist and primary care physician in Kazakhstan (2016-2024), the author repeatedly encountered patients whose cardiovascular profiles -- hypertension, ischemic heart disease, type 2 diabetes, chronic heart failure -- later preceded the development of significant cognitive impairment and dementia. The convergence of these conditions in the same patients, across years of clinical follow-up, reflects a trajectory that the mechanistic literature now confirms is not coincidental but pathophysiologically connected.

The Lancet Commission on dementia (2024) identified 14 modifiable risk factors responsible for approximately 45% of dementia cases, with vascular factors occupying the top tier [8]. The cardiovascular conditions managed daily in primary care are not merely comorbidities of aging -- they are active contributors to the neurodegeneration process. A screening framework that converts routine cardiovascular assessment into a first-stage filter for AD risk identification represents a direct clinical translation of this evidence.

Implementation considerations and limitations

Several implementation considerations must be acknowledged. The cardiovascular risk stratification at Stage 1 is not specific to AD -- elevated CRP, troponin, and NT-proBNP reflect broad vascular risk, and the positive predictive value of the composite for eventual AD pathology will require prospective calibration. The proposed thresholds for Stage 2 progression are derived from established cardiovascular practice guidelines, not from AD-specific evidence, and optimal thresholds for neurodegeneration risk prediction may differ.

P-tau217 assay standardization across platforms (Fujirebio Lumipulse, Lilly/Quanterix SIMOA, C2N ALZpath) remains an active area of development, and platform-specific reference ranges vary [6]. Centers for Medicare and Medicaid Services (CMS) national coverage determination for blood-based AD biomarker testing is pending, and the cost-effectiveness of the three-stage sequential approach at population scale will require formal health economic modeling.

This framework is proposed as a testable hypothesis requiring prospective validation in pilot studies across diverse primary care settings, with particular attention to populations with high cardiovascular risk burden and historically limited access to specialist neurology care.

## Conclusions

The patient population at highest cardiovascular risk and the population at highest AD risk are, to a substantial degree, the same people. They are already presenting to primary care physicians for cardiovascular management. Their biomarkers are already being measured. What is missing is the clinical framework that connects those biomarkers to the question of AD risk and routes the highest-risk patients toward blood-based biomarker confirmation testing while they are still in the pre-symptomatic window where disease-modifying treatment is most effective.

The three-stage integrated neuro-cardiological screening protocol proposed here makes several distinct contributions to the existing literature. First, it operationalizes the well-documented cardiovascular-neurodegenerative link into a concrete, stepwise clinical workflow deployable within existing primary care infrastructure -- an approach not previously described in the published literature. Second, it explicitly addresses the implementation gap identified by Burns et al., who documented that U.S. primary care physicians view P-tau217 testing as accurate and cost-effective but lack an operational pathway for systematic use. Third, by incorporating atrial fibrillation detection via ECG at Stage 2, the protocol captures one of the most recently confirmed independent risk factors for dementia at a stage when intervention remains possible. Fourth, the framework is specifically designed for populations with historically limited access to specialist neurology care -- a dimension of health equity that existing AD diagnostic pathways largely fail to address. Closing the AD diagnostic gap will not be achieved through a single breakthrough. It will require the systematic integration of existing clinical knowledge into practical, scalable screening frameworks. The cardiovascular biomarkers already measured in primary care may be the most accessible and most underutilized gateway to that goal.

## References

[1] Fertan E, Lam JY, Albertini G (2025). Lecanemab preferentially binds to smaller aggregates present at early Alzheimer's disease. Alzheimers Dement.

[2] Chen Y, Al-Nusaif M, Li S (2024). Progress on early diagnosing Alzheimer's disease. Front Aging Neurosci.

[3] (2026). Leqembi (lecanemab): U.S. Food and Drug Administration. FDA approves treatment for adults with Alzheimer's disease. https://www.fda.gov/drugs/news-events-human-drugs/fda-approves-treatment-adults-alzheimers-disease.

[4] (2026). Kisunla (donanemab): U.S. Food and Drug Administration. FDA approves treatment for adults with Alzheimer's disease. https://www.fda.gov/drugs/news-events-human-drugs/fda-approves-treatment-adults-alzheimers-disease-0.

[5] Jack CR Jr, Andrews JS, Beach TG (2024). Revised criteria for diagnosis and staging of Alzheimer's disease: Alzheimer's Association workgroup. Alzheimers Dement.

[6] Palmqvist S, Janelidze S, Quiroz YT (2020). Discriminative accuracy of plasma phospho-tau217 for Alzheimer disease vs other neurodegenerative disorders. JAMA.

[7] Burns JM, Alford S, Coppinger J, Jiménez-Mausbach M, Ray S, Pandey H, Laird R (2026). Early Alzheimer's diagnosis: U.S. primary care physicians and use of blood biomarkers. Alzheimers Dement.

[8] Livingston G, Huntley J, Sommerlad A (2024). Dementia prevention, intervention, and care: 2024 report of the Lancet standing Commission. Lancet.

[9] Orsucci D, Tessa A, Caldarazzo Ienco E (2024). Clinical and genetic features of dominant essential tremor in Tuscany, Italy: FUS, CAMTA1, ATXN1 and beyond. J Neurol Sci.

[10] Nedergaard M, Goldman SA (2020). Glymphatic failure as a final common pathway to dementia. Science.

[11] Smith AD, Refsum H (2016). Homocysteine, B vitamins, and cognitive impairment. Annu Rev Nutr.

[12] Dmytriw AA, Phan TG, Zerna C (2024). Atrial fibrillation and risk of dementia: a systematic review and meta-analysis. J Neurol Neurosurg Psychiatry.

